# Signal-Regulated Pre-mRNA Occupancy by the General Splicing Factor U2AF

**DOI:** 10.1371/journal.pone.0001418

**Published:** 2008-01-09

**Authors:** Anne Tisserant, Harald König

**Affiliations:** Forschungszentrum Karlsruhe GmbH, Institut für Toxikologie und Genetik, Karlsruhe, Germany; Lehigh University, United States of America

## Abstract

Alternative splicing of transcripts in a signal-dependent manner has emerged as an important concept to ensure appropriate expression of splice variants under different conditions. Binding of the general splicing factor U2AF to splice sites preceding alternatively spliced exons has been suggested to be an important step for splice site recognition. For splicing to proceed, U2AF has to be replaced by other factors. We show here that U2AF interacts with the signal-dependent splice regulator Sam68 and that forced expression of Sam68 results in enhanced binding of the U2AF65 subunit to an alternatively spliced pre-mRNA sequence *in vivo*. Conversely, the rapid signal-induced and phosphorylation-dependent interference with Sam68 binding to RNA was accompanied by reduced pre-mRNA occupancy of U2AF *in vivo*. Our data suggest that Sam68 can affect splice site occupancy by U2AF in signal-dependent splicing. We propose that the induced release of U2AF from pre-mRNA provides a regulatory step to control alternative splicing.

## Introduction

The split structure of most protein-encoding genes in eukaryotes requires the removal of non-coding sequences (introns) and the joining of coding parts (exons) during mRNA maturation by splicing [1]. By splicing precursor (pre-) mRNAs in different ways, multiple mRNAs and proteins can be generated from a single gene. Such alternative splicing appears as a major mechanism to expand the coding capacity of genomes [2], [3], and can explain the increase in organismal complexity during evolution, based on similar gene numbers [4], [5]. In vertebrates, at least 30–70% of genes give rise to alternatively spliced transcripts and splice variants play important roles during embryonic development and in other physiological processes [5], [6]. Furthermore, aberrant alternative splicing has been shown to underlie several pathological conditions in humans, including neurodegenerative disorders and cancer [3], [7].

Thus, appropriate generation of splice variants in the organism must be under the control of cues that determine their expression in different conditions. In fact, regulation of alternative splicing by extracellular signals has increasingly emerged as a mechanism for differential expression of gene products [8], [9]. Several cellular signaling pathways have been linked to splicing regulation. The targets of these pathways, how these targets are modulated, and how they are linked to the splicing machinery to regulate exon choice are key questions - though largely unresolved ones.

In many cases, selection of alternatively spliced exons has been shown to depend on regulatory RNA binding proteins. They interact with either the 5′ and 3′ splice sites or with regulatory sequences located in introns or exons, called splice silencer or enhancer sequences. These proteins affect binding of spliceosomal components to the splice sites and thus spliceosome formation [6]. A key step in spliceosome formation is the recognition of the polypyrimidine stretch and the terminal AG dinucleotide of the 3′ splice site by the 65 kDa and 35 kDa subunits, respectively, of the spliceosome component U2AF (U2 snRNP auxiliary factor) [10]. Alternatively spliced exons are often preceded by a weak polypyrimidine stretch (interrupted by purines) that shows weak affinity for U2AF65. Its binding to such weak sites has been suggested to be supported by the interaction with serine/arginine-rich (SR) splice enhancer proteins, that in some cases is dependent on U2AF35 [11]–[14].

We previously revealed the coupling of alternative splicing of variant exons of the cell surface molecule CD44 to the Ras-MAP-kinase pathway [15]. This evolutionary highly conserved signaling cascade regulates several physiological processes, including cell proliferation and survival, and its deregulation contributes to many cancers [16], [17]. The inclusion of variant (v) exons in CD44 mRNA occurs in some normal cells, e.g. in immune cells during activation, and in the course of the progression of various tumors. Certain CD44 variants were shown to be causally involved in metastasis formation [18]. By studying the Ras-pathway-stimulated splicing of one these variant CD44 exons (exon v5), we discovered the RNA-binding protein Sam68 as a splice regulator directly modified by this pathway in mouse T-lymphoma cells [19]. Sam68 can bind to sequences within the v5 exon and is phosphorylated by ERK MAP-kinase at several sites upon Ras-pathway activation. This phosphorylation is, at least in part, necessary for inclusion of the exon in CD44 RNA *in vitro* and *in vivo* [19].

Here we addressed the question of how the signal-dependent modification of the Sam68 protein can affect the splicing machinery. Our data suggest that Sam68 binds the general splicing factor U2AF and can affect its presence on pre-mRNA *in vivo*.

## Results

We previously identified Sam68 as a protein binding to sequences in the CD44 exon v5 and that regulates Ras-signaling-induced inclusion of the exon into mRNA. Furthermore, we showed that ERK-mediated phosphorylation of Sam68 is responsible for at least part of the Ras-induced inclusion of the exon [19]. Thus, Sam68 may modulate the activity of the splice-regulatory complex on the v5 exon to affect the recruitment of spliceosome components to the surrounding splice sites. Whereas the 5′ splice site downstream of the exon (GTAAG) corresponds to the consenus sequence (GTA/GAG), the preceding 3′ splice site is predicted to be weak. As for many alternatively spliced exons, it comprises an imperfect polypyrimidine tract interrupted by purines (Figure S1). It is thus predicted to be a weak target for U2AF65 binding [20], [21] and binding of U2AF65 could require support via protein-protein interactions from RNA-binding proteins regulating v5 splicing. In keeping with the idea that U2AF binding determines v5 exon inclusion, conversion to a consensus polypyrimidine stretch led to constitutive inclusion of the exon (Figure S1).

### Sam68 binds the general splicing factor U2AF

**Figure 1:**
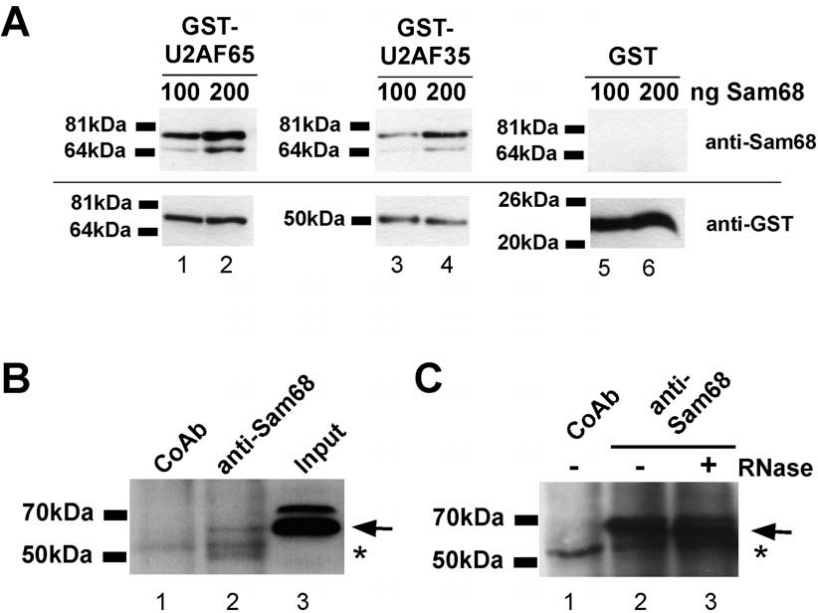
Sam68 interacts with U2AF *in vitro* and *in vivo*. (A) Immunoblot analysis of GST pull-down reactions involving recombinant Sam68 and GST-fusion proteins of U2AF65 and U2AF35. The GST protein served as a control. Immunodetection with anti-Sam68 and anti-GST antibodies is shown in the upper and lower panels, respectively. Black bars on the left indicate positions of marker bands. (B) Co-immunoprecipitation from LB17-lymphoma cell lysates with an anti-Sam68 antibody or a corresponding control antibody (CoAb). In lane 3, the lysate was loaded. Precipitates were analysed by immunoblotting with an anti-U2AF65 antibody. Arrows indicate U2AF65 band; asterisks denote bands caused by immunoglobulins. (C) Co-immunoprecipitation experiment as in (B), in the presence (+) or absence (−) of RNase A. Symbols are as in (B).

To test the possibility that Sam68 could affect U2AF binding by protein-protein interaction, we initially tested whether the two proteins can interact *in vitro*. Therefore, we performed pull-down experiments with bacterially expressed Glutathione S-transferase (GST) fusion proteins of U2AF65 and U2AF35, respectively, and recombinant His-tagged Sam68. As shown in Figure 1A by immunoblotting with an anti-Sam68 antibody, both subunits of U2AF precipitated Sam68 in a dose-dependent manner (lanes 1–4). This was not the case when we performed the assays with the GST portion alone (lanes 5–6), suggesting U2AF-dependent interaction. To investigate whether Sam68 could be complexed with U2AF *in vivo*, we performed co-immunoprecipitations with an anti-Sam68 antibody and lysates from LB17 mouse lymphoma cells. Immunoblotting with an anti-U2AF65 antibody showed that the anti-Sam68 precipitate contained U2AF65 (Figure 1B, compare lanes 2 and 3). Precipitates from the control antibody did not show the U2AF65 band (Figure 1B, lane 1), indicating Sam68-dependent precipitation of the protein. Treatment of the lysates prior and during precipitation by RNase A did not interfere with co-precipitation of U2AF65 (Figure 1C, compare lanes 2 and 3), pointing to the association of the proteins independent of RNA bridging. Due to the poor sensitivity of the U2AF35 antibody available, we could not test the presence of U2AF35 in the precipitates. Taken together these data suggest that Sam68 can directly interact with U2AF proteins and that Sam68 is associated with U2AF65 *in vivo*.

Ras-signaling-induced inclusion of CD44 exon v5 in mRNA is at least partially dependent on phosphorylation of Sam68 by ERK [19]. Since U2AF is a critical factor in splice site selection and given the interaction of Sam68 and U2AF, we wished to test the possibility that the signaling-induced phosphorylation of Sam68 is linked to splicing by affecting its interaction with U2AF. Unfortunately only a small proportion of Sam68 can be obtained in its phosphorylated form upon Ras-pathway activation in cell lysates [19], so that we could not test the binding of phosphorylated Sam68 to U2AF in co-immunoprecipitations. Therefore, we performed pull-down experiments with GST-fusions of U2AF65 and U2AF35, and recombinant Sam68 that was either left unphosphorylated or had been phosphorylated by ERK *in vitro*. In these experiments, we could detect no effect of Sam68 phosphorylation on the interaction of the proteins (data not shown).

### Phosphorylation of Sam68 interferes with binding to its cognate RNA elements

We then wished to test whether phosphorylation of Sam68 by ERK could instead affect its binding to splice-regulatory RNA elements.

**Figure 2:**
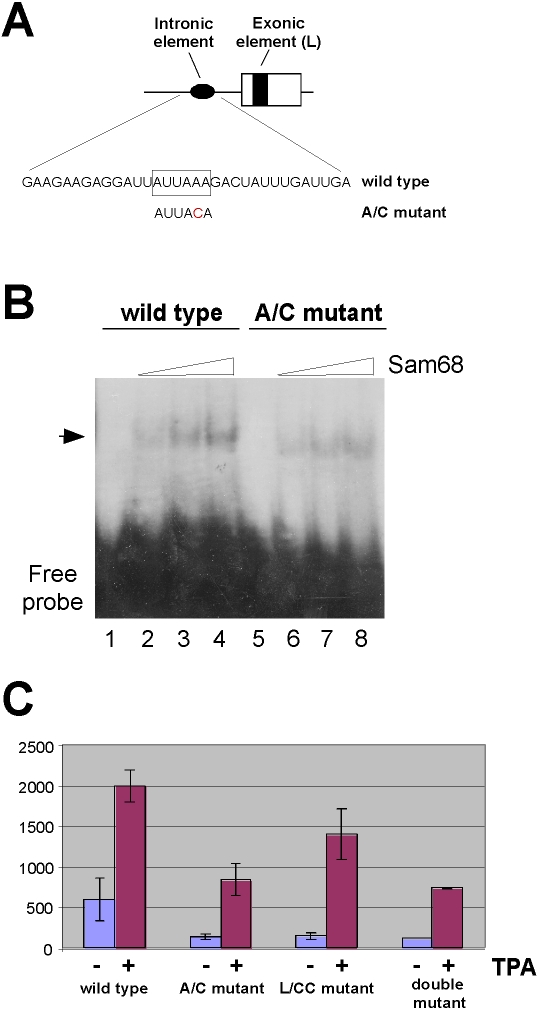
Intronic and exonic Sam68 binding sites affect CD44 v5 exon inclusion. (A) Scheme indicating positions of the Sam68 binding sites (black oval and box, respectively) in CD44 exon v5 (open box) and the upstream intron (line). The Sam68 consensus binding site in the wild-type RNA sequence used for the electrophoretic mobility shift assay (EMSA) in panel B is boxed. The mutated nucleotide of the A/C mutant is indicated in red. (B) EMSA using recombinant Sam68 and radioactively labeled RNA oligonucleotide probes comprising either the wild-type or the A/C-mutant version of the intronic Sam68 binding site (see panel A). (C) v5-luciferase fusion activity from LB17 lymphoma cells transfected with different pETv5luc splice-reporter genes [15]. They were mutated for either the exonic (L/CC mutant [19]), the intronic (A/C mutant), or both Sam68 binding sites (double mutant). Cells were treated with 12-o-tetradecanoylphorbol-13-acetate (TPA, 40 ngml^−1^) (+) or with DMSO (solvent control) (−) for 6h prior to lysis and measurement of luciferase activity. Error bars indicate standard deviations from three independent transfections.

**Figure 3:**
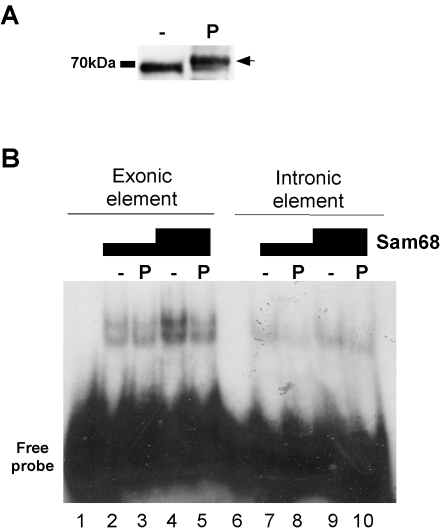
Phosphorylation of Sam68 by ERK MAP-kinase impairs its binding to RNA *in vitro*. (A) Immunoblotting of non-phosphorylated (−) and ERK-phosphorylated (P) recombinant Sam68 preparations used in (B). Phosphorylation of Sam68 at ERK target sites results in a molecular weight shift (arrow) [19]. Black bar on the left indicates marker band. (B) Electrophoretic mobility shift assay using RNA oligonucleotide probes comprising the exonic or intronic Sam68 binding sites and different amounts of either non-phosphorylated (−) or phosphorylated (P) recombinant Sam68.

A Sam68-binding sequence in CD44 exon v5 was shown to determine the overall level of exon inclusion [19]. We found a second site corresponding to the Sam68 consensus binding sequence [22] in the intron preceding the v5 exon, immediately upstream of the putative branch point sequence (Figure 2A). To test whether this second site can indeed bind Sam68, we performed electrophoretic mobility shift assays (EMSA) involving recombinant Sam68 and RNA oligonucleotides comprising this site or a point-mutated version of it (A/C mutant; see Figure 2A). This mutation should interfere with Sam68 binding [22]. As shown in Figure 2B, the RNA oligonucleotide comprising the wild-type sequence bound Sam68 in a dose-dependent manner (lanes 1–4), whereas binding to the A/C-mutated version was severely compromised (lanes 5–8), indicating that Sam68 binds to the oligonucleotide depending on this sequence. To test the functional relevance of this intronic Sam68 binding-element, we introduced the A/C mutation into a luciferase-based splice-reporter minigene carrying CD44 exon v5 [15] and transfected it into LB17 mouse lymphoma cells. In these cells inclusion of the v5 exon in mRNA can be induced by Ras-pathway activation upon phorbol-ester treatment [15], [23]. Similar to the mutation of the exonic Sam68 binding site [19], the A/C mutation of the intronic Sam68 reduced the overall level of v5 exon inclusion, whereas induction of exon inclusion by phorbol ester treatment was still functional (Figure 2C). The same was true for a construct in which both Sam68 binding sites were mutated (Figure 2C). These results suggest that binding of Sam68 to these elements in the CD44 pre-mRNA determines the overall level of v5 exon inclusion in mRNA. Signal-mediated induction of splicing, although dependent on Sam68 [19], seems however not to depend on these sites. Could there still be a link between Sam68 bound to these RNA elements and splicing induction? To address this question, we initially performed electrophoretic mobility shift assays (EMSA) employing RNA oligonucleotides comprising either of the two Sam68 binding elements and different amounts of either non-phosphorylated Sam68 or of Sam68 thiophosphorylated by ERK *in vitro* (Figure 3). The stable thiophosphorylation results in the same shift in molecular weight (see Figure 3A) as compared to normal phophorylation and is active in inducing v5 exon splicing *in vitro* [19]. So phosphorylated Sam68 consistently shifted less radioactively labeled oligonucleotide as compared to non-phosphorylated Sam68, both with the exonic element (Figure 3B, lanes 2–5) and the intronic element probe (Figure 3B, lanes 7–10). This result suggests that phosphorylation of Sam68 by ERK interferes with the ability of Sam68 to bind its cognate RNA binding elements *in vitro*.

### Signaling-induced inhibition of Sam68 binding to pre-mRNA *in vivo*

**Figure 4:**
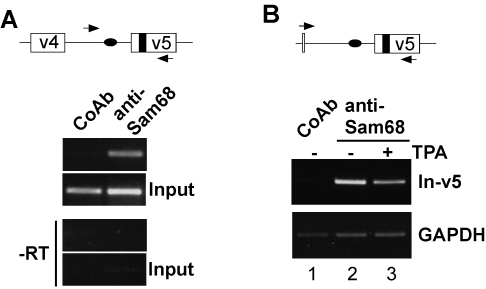
Signaling-induced interference with Sam68 binding to pre-mRNA *in vivo*. (A) Detection of Sam68 binding to an endogenous CD44 pre-mRNA fragment (spanning the v5 exon and 100 bp upstream intron sequence) in LB17 lymphoma cells by RNP immunoprecipitations. A control antibody (CoAb) or an anti-Sam68 antibody were used for precipitations. Lower panels show amplifications from the lysate used as input for the precipitations. Bands correspond to the expected size of 228 bp. Positions of primers used for amplification are indicated as arrowheads in scheme on top; Sam68 binding sites (black oval and box, respectively), CD44 exons v4 and v5 (open boxes), intron (line). –RT represents reactions without reverse transcriptase. (B) RNP immunoprecipitations with either a control antibody (CoAb) or an anti-Sam68 antibody from lysates of LB17 lymphoma cells that were transfected with the pETv5 minigene construct [23]. The cells were either left untreated (−) or treated with 40 ng/ml of 12-o-tetradecanoylphorbol-13-acetate (TPA) for 10 min. A fragment from the minigene pre-mRNA spanning the v5 exon and 450 bp upstream intron sequence (In-v5) was amplified using the primers indicated (scheme on top; symbols as in panel A). Specific amplification of the minigene pre-mRNA is achieved by the forward primer that is complementary to the deletion junction of the v4 exon in the construct. To check for potential differential loss of material during the precipitation and washing procedure, we monitored the amounts of GAPDH mRNA unspecifically bound to the antibody and/or the agarose beads (lower panel). Bands correspond to the expected sizes of 600 bp (In-v5) and 278 bp (GAPDH). All PCR amplifications were in linear phase as verified with different amounts of cDNA.

Based on these results one would postulate that stimulation of the Ras-Erk MAP-kinase cascade results in less Sam68 bound to pre-mRNA carrying such elements. To test this hypothesis *in vivo*, we adapted the ribonucleoprotein (RNP) immunoprecipitation technique [24] to determine the occupancy of the corresponding CD44 pre-mRNA region by Sam68. Analogous to the detection of DNA-protein interactions in chromatin immunoprecipitation, this method detects RNA-protein interactions *in vivo*. Employing reversible formaldehyde crosslinking and high-stringency immunoprecipitation, specific RNA fragments associated with a given protein are identified by reverse transcription followed by PCR (RT-PCR). We initially showed that a fragment from endogenous CD44 pre-mRNA spanning the v5 exon and 110 nucleotides of the upstream intron sequence could be amplified from precipitates of an anti-Sam68 antibody, but not from precipitates of a corresponding control antibody (Figure 4A). This result indicates specific precipitation of the RNA fragment and suggests the occupancy of this CD44 pre-mRNA region by Sam68 *in vivo*. Since only a small proportion of endogenous CD44 pre-mRNA molecules include CD44 exon v5 upon phorbol-ester stimulation in our lymphoma cell model [23], changes in protein binding to this minority of molecules would be difficult to detect. To overcome this issue, we transiently transfected the LB17 lymphoma cells with a minigene carrying the v5 exon and surrounding intron sequences, flanked by insulin exons (pETv5). Upon phorbol-ester treatment, the majority of pre-mRNA molecules from this construct include the v5 exon [23]. As shown in Figure 4B, the anti-Sam68 antibody specifically precipitated a fragment spanning the v5 exon and the upstream intron sequence from the minigene pre-mRNA (compare lanes 1 and 2). Phorbol-ester treatment of the cells for as little as ten minutes, which induces ERK-mediated Sam68 phosphorylation ([19] and data not shown), consistently led to a marked decrease in the amount of precipitated fragment (lanes 2 and 3). This result indicates interference with the occupancy of the RNA fragment by Sam68. To further ascertain that the differences seen between the samples are not caused by differential loss of precipitation material during handling, we wished to amplify traces of RNA bound unspecifically to the antibody and/or the agarose beads used for precipitation. Therefore, we amplified a fragment of GAPDH mRNA. This fragment was present at similar amounts in the different samples, including the control antibody sample (Figure 4B, lower panel), indicating no differential loss of material between the specimens.

Taken together, the results on RNA binding obtained *in vitro* and *in vivo* suggest that the Ras-signaling-induced phosphorylation of Sam68 by ERK interferes with Sam68 binding to its elements on pre-mRNA.

### Sam68 and regulated pre-mRNA occupancy by U2AF

**Figure 5:**
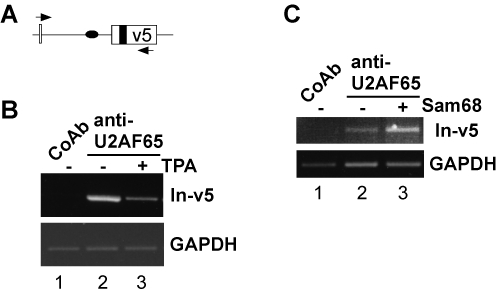
Sam68 and pre-mRNA occupancy by U2AF. (A) Schematic drawing of the examined CD44 minigene pre-mRNA region (CD44 exon v5, open box; upstream intron, line; Sam68 binding sites, black oval and box, respectively; arrows, PCR primer). (B) RNP immunoprecipitations with an anti-U2AF65 antibody or a control antibody (CoAb) from lysates of LB17 lymphoma cells that were transfected with the pETv5 minigene construct containing CD44 exon v5. Cells were left either untreated (−) or treated with phorbol ester (TPA). TPA treatment and RNP immunoprecipitations were performed as described in legend to Figure 4. (C) RNP immunoprecipitations of U2AF65 from LB17 lymphoma cells co-transfected with the pETv5 minigene construct and either a Sam68 expression plasmid (+) or the empty expression vector as a control (−). Bands correspond to the expected sizes of 600 bp (In-v5) and 278 bp (GAPDH). All PCR amplifications were in linear phase as verified with different amounts of cDNA.

Given the interaction of Sam68 and U2AF65, binding of Sam68 might stabilize association of U2AF with pre-mRNA. If this were true, the rapid release from RNA of Sam68 upon phorbol-ester treatment of the cells, should result in decreased pre-mRNA occupancy by U2AF65 *in vivo*. To test this prediction, we performed RNP immunoprecipitation experiments using an anti-U2AF65 antibody. We could specifically precipitate RNA fragments containing the CD44 v5 exon and the upstream intron sequence (Figure 5A, lanes 1 and 2). Furthermore, phorbol-ester treatment of the cells for ten minutes led to a marked reduction of the fragments detectable in the U2AF immunoprecipitate (Figure 5A, compare lanes 2 and 3). This finding supports the idea that RNA-bound Sam68 can act as a stabilizing factor for U2AF binding. To test this idea further, we examined if forced expression of Sam68 can increase the occupancy of this pre-mRNA region by U2AF65. Figure 5C shows that co-transfection of a Sam68 expression vector led to an increase in the RNA fragments precipitated with the anti-U2AF65 antibody when compared to control-vector transfected cells (lanes 2 and 3). Together with the GST pull-down and co-immunoprecipitation data, these findings point to Sam68 as a factor that can stabilize U2AF binding by protein-protein interaction *in vivo*. Moreover, they suggest that Ras-signaling-induced phosphorylation of Sam68 interferes with binding of Sam68 to RNA, and may thus affect splice site occupancy by U2AF65.

## Discussion

The binding of U2AF65 to the pyrimidine stretch of the 3′ splice site is a crucial step for exon definition and spliceosome assembly in higher eukaryotes [6], [10]. Alternatively spliced exons are often preceded by a poor pyrimidine tract and their splicing is dependent on exonic splice enhancers elements [11], [12]. For some of these exons, biochemical experiments have suggested the recruitment of U2AF through interaction of its 35 kDa unit with serine/arginine-rich (SR) domains of splice enhancer factors (U2AF recruitment model) [13]. Notably, U2AF65 binds the pre-mRNA only transiently in the course of spliceosome formation. It dissociates from pre-mRNA in the first ATP-dependent pre-spliceosome complex, followed by further spliceosome assembly [25], [26].

Our data suggest that U2AF interacts with the signal-dependent splice regulator Sam68 by protein-protein interaction and that Sam68 can support binding of U2AF to pre-mRNA *in vivo*. The two proteins appear to be part of a preformed, signal-responsive complex over the CD44 v5 exon and the adjacent upstream intronic sequences. The binding of Sam68 to its two binding sites would be necessary for efficient complex formation, involving the recruitment of U2AF, and so could determine the overall level of v5 exon inclusion. Concomitantly, Sam68 is a factor targeted and phosphorylated through the Ras-MAP-kinase signaling cascade, a step necessary for the signal-induced splicing [19] and that results in its release from RNA (this work). The rapid signal-dependent decrease in Sam68 binding to pre-mRNA could allow to reorganize the complex (that may still contain Sam68 but without contacting RNA anymore) and thus initiate the induction process of v5 exon splicing. However, given that the Sam68 binding-site mutants are still inducible (see Figure 2C), Sam68 phosphorylation appears to affect also other properties of the protein besides RNA binding, conceivably as a result of a conformational change.

Interestingly, the signal-induced release of Sam68 from RNA was paralleled by decreased U2AF65 occupancy of corresponding pre-mRNA fragments *in vivo*. Together with the data showing that phosphorylation of Sam68 by ERK MAP-kinase down-regulates the ability of Sam68 to bind its cognate RNA elements, the results suggest that Sam68 may so affect splice site occupancy of its binding partner U2AF in a signal-dependent manner. This should result in reorganization of factor binding to the 3′ splice site and could allow further spliceosome assembly. Our data do not exclude that U2AF65 itself may be an additional target for signal-dependent modification of its binding to RNA. Thus, the findings link the release of U2AF from pre-mRNA to a signaling cascade that activates splicing in response to extracellular cues. The *in vivo* kinetics of splicing has been suggested to be very rapid, at least for efficient constitutive splicing events, showing halflives of 0.4–7.5 minutes [27]. We thus cannot exclude that the release of U2AF from the corresponding CD44 pre-mRNA region within minutes, observed here, is a consequence rather than the cause of signal-induced splicing. However, our data on the interaction of the two proteins, and on U2AF binding to pre-mRNA supported by Sam68 *in vivo*, suggest that it is part of the Sam68-dependent induction process. The requirements for the release or displacement of U2AF from splice sites during spliceosome assembly are only poorly understood. It should however depend on the dynamic interaction with other proteins, like spliceosome-associated proteins (SAPs) and U5 snRNP proteins, that appear to replace it during further spliceosome assembly [25], [26], or splice enhancer proteins also involved in constitutive splicing. Thus, the induced release of U2AF from pre-mRNA involving signal-responsive partners like Sam68 could be a step to control spliceosome assembly at regulated splice sites.

## Materials and Methods

### Cell culture and transfections

LB17 2.3 murine T-lymphoma cells were grown as described [23], [28]. Transient transfections with the pETv5 minigene construct [23] were performed with the polycationic SuperFect reagent (Qiagen) according to the instructions of the manufacturer, using 5 µg of DNA. For Sam68 overexpression, 3 µg of pETv5 were cotransfected with with 2 µg of either pcDNA3-Sam68 [19] or of the empty vector as a control.

### GST pull-down assay

0.5–1 µg of GST-U2AF fusion proteins or of GST as a control were incubated with 100 or 200 ng of recombinant His-Sam68 in 50 µl binding buffer (10 mM HEPES pH 7.9, 150 mM KCl, 0.1% NP40, 0.5 mg/ml BSA, 1 mM DTT, 1 mM PMSF) at 30°C for 1 h. 500 µl binding buffer containing 20 µl of a 50% slurry of glutathione sepharose were added and reactions were rotated at room temperature (RT) for 90 min. After washing three times with binding buffer, precipitates were analysed by SDS-PAGE and immunoblotting with the anti-Sam68 antibody C20 or the anti-GST antibody Z5 (both from Santa Cruz Biotechnology). GST-fusion proteins of U2AF65 and U2AF35 were expressed in E.coli BL21 from constructs carrying corresponding human cDNAs (starting with the second codon) in the Xho I site of pGEX-4T-3 (Amersham Pharmacia). Fusion proteins were purified by the GST-purification module (Amersham Pharmacia) according to the instructions of the manufacturer. His-Sam68 was obtained as described below.

### Co-immunoprecipitations

Cells were lysed in CoIP buffer (50 mM Tris-HCl pH 8.0, 100 mM NaCl, 0.5% NP40; supplemented with 0.1 mM DTT; 1 mM PMSF; 2 µg/ml Aprotinin and 2 µg/ml Leupeptin) and the cleared lysate was incubated with a polyclonal rabbit anti-Sam68 antibody (C20, Santa Cruz Biotechnology) bound to protein A/G agarose at 4°C for 1h. For RNase treatment, 0.2 µg/µl RNase A was added to the lysates 60 min prior to precipitation. Precipitates were washed three times with CoIP buffer. After SDS-PAGE, U2AF65 was detected by immunoblotting with a mouse monoclonal anti-U2AF65 antibody (clone MC3; Sigma-Aldrich).

### RNP immunoprecipitation

1–2×10^7^ cells were used for *in vivo* crosslinking using 0.1% formaldehyde and lysed as described [24]. Crosslinked complexes were solubilized and RNA was fragmented to a length of 400–600 nucleotides by sonification. Immunoprecipitations were performed with protein A/G agarose coated with 2 µg of either the polyclonal rabbit anti-Sam68 antibody C20 (Santa Cruz Biotechnology) and a polyclonal rabbit anti-GST antibody as a control, or with a monoclonal mouse anti-U2AF65 antibody (MC3, Sigma-Aldrich) and mouse IgG as a control, at RT for 90 min. Prior to immunoprecipitation, protein A/G was incubated with 20 units of RNasin (Promega) for 10 min. Immunoprecipitates were washed four times with high-stringency RIPA buffer (50 mM Tris-HCl pH 7.5, 1 M NaCl 1% NP40, 1% sodium deoxycholate, 0.1% SDS, 1 mM EDTA, 1 M Urea, 0.2 mM PMSF). Precipitated complexes were de-crosslinked in 100 µl 50 mM Tris-HCl pH 7.0, 1% SDS, 5 mM EDTA, 10 mM DTT at 70°C for 45 min and RNA was extracted using RNAPure (Peqlab) followed by DNase digestion. Half of the RNA was reverse transcribed involving random priming. For amplification of the endogenous CD44 pre-mRNA fragment (228 bp in length), primers hybridizing 60 bp upstream of the intronic Sam68 binding site (forward primer; GCTTAGGCATAGAACCAGTAGA) and at the end of CD44 exon v5 (reverse primer; TACTTGTGCTTGTAGCATGTGG) were used. For detecting the pre-mRNA fragment from the pETv5 minigene (607 bp in length), a primer spanning the deletion site of CD44 exon v4 was used as forward primer (TCATTATGACAGTTGCCACCAG). Thirty and thitry-five PCR cycles (95°C for 1 min; 55°C for 1 min; and 72°C for 1.5 min) were performed for amplification of fragments from the endogenous CD44 gene and the transfected minigene, respectively. PCRs for GAPDH (278 bp) were performed using the primers AGACAGCCGCATCTTCTTGTGC and CTCCTGGAAGATGGTGATGG. Thirty cycles (95°C for 1 min; 55°C for 1 min; and 72°C for 1.5 min) were performed.

### Electrophoretic mobility shift assay (EMSA)

EMSA reactions were performed as described [19] using radioactively end-labeled RNA oligonucleotides and recombinant non-phosphorylated His-Sam68 or His-Sam68 phosphorylated with ERK MAP-kinase *in vitro*. Oligonucleotide sequences comprising the exonic and intronic Sam68 binding sites were AUAGACAGAAUCAGCACCAGUGCUCAUGGAGAAAAUUGGACC and GAAGAAGAGGAUUAUUAAAGACUAUUUGAUUAGA, respectively. His-tagged Sam68 was expressed from E.coli strain M15 from a pQE-32 construct (Qiagen) carrying the mouse Sam68 cDNA sequence. Purification of the protein was performed using NI-NTA agarose (Qiagen). *In vitro* phosphorylation by ERK in the presence of 1 mM ATP-γS was performed by four consecutive reaction cycles using 200 units of activated recombinant ERK2 (New England Biolabs) as described [19]. We used 25 and 50 ng recombinant Sam68 in the EMSA with the exonic probe and 175 and 250 ng with the intronic probe.

## Supporting Information

Figure S1 (0.14 MB DOC)
