# Supplementary material for: Signal-Regulated Pre-mRNA Occupancy by the General Splicing Factor U2AF
Source: PLoS One. 2008 Jan 9;3(1):e1418. doi: 10.1371/journal.pone.0001418 (PMC2169300; doi:10.1371/journal.pone.0001418)
Supplement: Figure S1 — (0.14 MB DOC) [file pone.0001418.s001.doc]

**Supporting information**

**
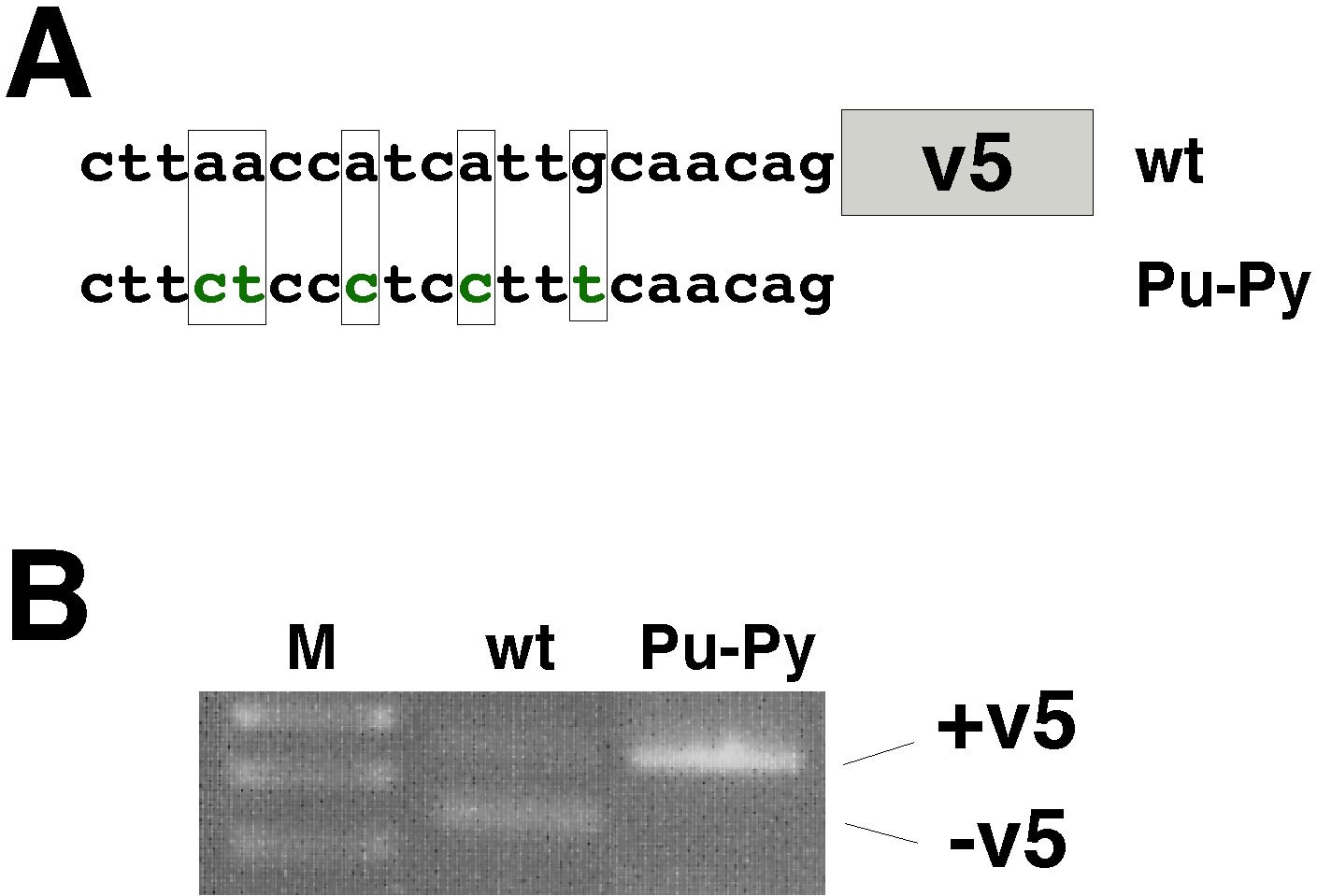
**

**Figure S1**

**Conversion to a consensus polypyrimidine stretch results in enhanced splicing of CD44 v5.** (**A**) Scheme indicating the sequences of the wild-type (wt) and purine-to-pyrimidine converted (Pu-Py) pyrimidine stretch preceding CD44 exon v5 (grey box). Converted nucleotide positions are boxed, converted nucleotides are in green. (**B**) RT-PCR analysis of exon-v5 containing minigene constructs harboring the wild-type or the converted polypyrimidine tract. The minigene plasmids were transiently transfected into murine LB17 lymphoma cells. RNA was analyzed 24h after transfection.
